# Selecting human papillomavirus genotypes to optimize the performance of screening tests among South African women

**DOI:** 10.1002/cam4.3329

**Published:** 2020-07-24

**Authors:** Lauren G. Johnson, Rakiya Saidu, Zizipho Mbulawa, Anna‐Lise Williamson, Rosalind Boa, Ana Tergas, Jennifer Moodley, David Persing, Scott Campbell, Wei‐Yann Tsai, Thomas C. Wright, Lynette Denny, Louise Kuhn

**Affiliations:** ^1^ Department of Epidemiology Mailman School of Public Health Columbia University Irving Medical Center New York NY USA; ^2^ School of Nursing Columbia University Irving Medical Center New York NY USA; ^3^ Department of Obstetrics and Gynecology University of Cape Town Cape Town South Africa; ^4^ South African Medical Research Council Gynaecologic Cancer Research Centre (SAMRC GCRC) University of Cape Town Cape Town South Africa; ^5^ Department of Pathology Institute of Infectious Disease and Molecular Medicine University of Cape Town Cape Town South Africa; ^6^ Department of Laboratory Medicine and Pathology National Health Laboratory Services Walter Sisulu University Mthatha South Africa; ^7^ Department of Obstetrics and Gynecology Vagelos College of Physicians and Surgeons Columbia University Irving Medical Center New York NY USA; ^8^ Women’s Health Research Unit School of Public Health and Family Medicine University of Cape Town Cape Town South Africa; ^9^ Cepheid Sunnyvale CA USA; ^10^ Department of Biostatistics Mailman School of Public Health Columbia University Irving Medical Center New York NY USA; ^11^ Department of Pathology Vagelos College of Physicians and Surgeons Columbia University Irving Medical Center New York NY USA; ^12^ Gertrude H. Sergievsky Center Vagelos College of Physicians and Surgeons Columbia University Irving Medical Center New York NY USA

**Keywords:** HPV genotyping assays, human papillomavirus, sensitivity, South Africa, specificity

## Abstract

Human papillomavirus (HPV) testing is highly sensitive compared to cytology, with the trade‐off of being less specific. We investigated whether select combinations of HPV genotypes, ascertained by Linear Array (LA) and Xpert HPV (GX), can optimize sensitivity/specificity trade‐offs to detect high‐grade cervical intraepithelial neoplasia (CIN2+). In a study in Cape Town, South Africa, 586 women living without and 535 living with HIV, aged 30‐65 years, were recruited. Each woman underwent a pelvic exam to collect cervical samples (tested by LA and GX for 14 high‐risk HPV genotypes) and underwent colposcopy with histological sampling to determine CIN2+. In multivariable logistic regression of LA results, only HPV genotypes 16, 18, 31, 33, 35, 52, 58 were significantly associated with CIN2+ (*P* < .05). Xpert includes these seven types along with HPV 45 within three of the test's five channels and we defined these eight types as restricted genotyping (ie 16, 18, 31, 33, 35, 45, 52, 58). Full genotyping was defined as all 14 high‐risk types. Sensitivity estimates for full genotyping using LA were similar to that of restricted genotyping: 83.9% (full) vs 79.0% (restricted) in women without HIV and 93.0% (full) vs 88.9% (restricted) in women living with HIV. Specificity estimates improved for restricted vs full genotyping: 87.4% (full) vs 90.8% (restricted) in women without HIV and 63.7% (full) vs 71.4% (restricted) in women living with HIV. To optimize the performance of HPV testing for cervical cancer screening in high‐burden, under‐resourced settings like South Africa, only HPV 16, 18, 31, 33, 35, 45, 52, 58 could be included to define screen‐positive. We recommend the inclusion of HPV45 for its known link to adenocarcinoma.

## Graphical abstract

Human papillomavirus (HPV) testing is highly sensitive compared to cytology, with the trade‐off of being less specific. Among South African women, we have shown that restricting the definition of screen‐positive to women with HPV genotypes 16/18/45/31/33/35/52/58 substantially improves the specificity of screening while producing only minor reductions in sensitivity compared to inclusion of all 14 HPV genotypes conventionally included as high risk in HPV assays.

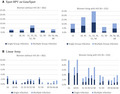

## 1 INTRODUCTION

HPV primary screening is increasingly being considered as the preferred approach for cervical cancer screening in many countries with established screening programs.^1^ HPV primary screening also provides a viable option for high‐burden, low‐resource settings, especially since affordable, point‐of‐care assays are now available.^2^, ^3^, ^4^ Point‐of‐care tests are conducted in the clinic setting with non‐physician providers and produce results within the same day, alleviating the burden of limited laboratory infrastructure and personnel.^5^ Furthermore, HPV DNA testing has been demonstrated to be considerably more sensitive than cytology^6^, ^7^ and is more sensitive, specific, and reproducible than the visual inspection with acetic acid (VIA).^8^ The low sensitivity of cytology necessitates frequent, repeated screening to be effective, making it susceptible to high attrition and costly follow‐up.^2^ VIA is a subjective, visual skill that requires intensive quality assurance monitoring and frequent refresher courses to ensure test performance does not fluctuate by provider.^3^, ^5^, ^8^

Although advantageous in many regards, HPV DNA testing has the trade‐off of having relatively low specificity,^7^, ^9^, ^10^, ^11^ particularly in women living with HIV.^5^, ^12^ In the screen‐and‐treat model, now widely recommended as the most appropriate approach for low‐resource settings, the relatively low specificity of HPV DNA testing means that sizable numbers of women may be overtreated for transient infections that will ultimately regress.^13^ Scarce resources would be unnecessarily spent on women at low risk for CIN2+ being referred for further colposcopy follow‐up or treatment.^14^ A potential option for reducing the risk of overtreatment is triaging HPV‐positive women with VIA or cytology screening,^15^, ^16^, ^17^, ^18^, ^19^, ^20^ which guidelines from WHO and ASCO have outlined.^13^, ^21^ However, both of these approaches have limitations and other alternatives would be desirable.

Here we evaluate whether the strategy of limiting the number of high‐risk HPV genotypes included in HPV screening assays could improve the clinical performance of HPV testing. Genotyping for specific HPV genotypes could provide risk stratification of HPV‐positive women by identifying HPV infections that are at highest risk of persisting and developing into invasive cervical cancer.^20^, ^22^ Of the approximately 40 HPV genotypes that affect the anogenital tract, 14 types (16, 18, 31, 33, 35, 39, 45, 51, 52, 56, 58, 59, 66, 68) are considered high‐risk for cervical cancer but vary in their degrees of oncogenicity.^23^ HPV 16 and 18 are universally accepted as being the most carcinogenic (present in ~70% of invasive cancers).^24^, ^25^, ^26^ However, there is less consensus surrounding the relative importance of the other high‐risk genotypes.^22^ Based on the results from genotyping over 12 000 invasive anogenital cancers, the relative contribution of HPV 16, 18, 31, 33, 45, 52, 58 to female anogenital cancer was found to be approximately 90% and, based on this, these are the high‐risk genotypes incorporated in the nanovalent HPV vaccine.^27^ This suggests that restricted genotyping could be effectively utilized to triage HPV‐positive women in the context of screening.

In this study, we evaluated the impact on sensitivity and specificity for CIN2+ of restricting the number of HPV genotypes identified using the Roche Linear Array (LA) method that identifies each of the high‐risk HPV genotypes individually. We also compared the individual LA results to the grouped HPV typing inherent in the Xpert HPV (GX) assay.

## 2 METHODS AND MATERIALS

### 2.1 Setting and population

We recruited women from two populations in Cape Town, South Africa. One was a community‐based clinic (screening population) located in Khayelitsha township which provides general women's health services to the local community. The other was women referred to a colposcopy clinic at Groote Schuur Hospital, a university teaching hospital, for evaluation of abnormal cervical cytology (referral population). Women aged 30‐65 years were eligible to participate if they: (a) had a known and documented HIV status, (b) were not previously treated for cervical disease, (c) did not have a hysterectomy, and (d) were not pregnant. The study aimed to recruit approximately equal numbers of women living with and without HIV. Seven hundred and fifteen women were recruited at the community‐based clinic and 406 from the referral colposcopy clinic (total of 1121 women).

### 2.2 Study procedures

This study was approved by institutional review boards at Columbia University (AAAO3652) and the University of Cape Town. Following written informed consent, each woman underwent a pelvic examination by a physician and had two liquid‐based cytology specimens (ThinPrep Pap Test, Hologic) collected using a cytobrush and plastic spatula for each (Medscand).

After the collection of the cervical samples, a colposcopic examination was performed. Multiple cervical biopsies were obtained of the most abnormal‐appearing areas of the cervix and/or an endocervical curettage (ECC) if there was no visible cervical lesion. If clinically indicated based on clinical protocol, a Loop Electrosurgical Excision Procedure (LEEP) was performed. Women from the screening population were told to return in 6 weeks after enrollment to receive their histology results. For those women recruited at the screening clinic, a second colposcopy was undertaken in those with HPV at baseline who had not had cervical intraepithelial neoplasia grade 2 or greater (CIN2+) detected at baseline. A third colposcopy was undertaken if the second colposcopy still failed to identify CIN2+. Random four‐quadrant biopsies were undertaken at these repeat colposcopies to reduce risks of missing disease.

Histological samples collected via biopsy, LEEP, and/or ECC were first evaluated by a pathologist in Cape Town and then blindly reviewed by an expert pathologist in the US. Results were classified using the cervical intraepithelial neoplasia (CIN) classification system: within normal limits (WNL), CIN grade 1 (CIN1), CIN grade 2 (CIN2), CIN grade 3 (CIN3), and cancer.^28^ Any discrepancies were resolved by a third pathologist as a tie‐breaker to reach a final consensus diagnosis.

### 2.3 HPV testing

One mL of the first cervical sample was tested at the Khayelitsha screening clinic using the Xpert HPV assay (Cepheid). The remaining 19 mL were stored and tested later in batches at the University of Cape Town using Linear Array (LA) (Roche Diagnostics). The second sample was stored for possible later testing.

Xpert HPV (GX) is a clinic‐based, point‐of‐care HPV assay that tests for 14 high‐risk HPV types in five separate channels through real‐time PCR. Each single‐use Xpert HPV cartridge is prefilled with the necessary reagents, primers, and fluorescent probes to amplify the E6 and E7 regions of HPV DNA automatically on the GeneXpert System (Cepheid). Presence of HPV DNA that meets cycle thresholds is indicated by fluorescence in the following five channels: HPV16; HPV18, 45; HPV31, 33, 35, 52, 58; HPV51, 59; and HPV39, 56, 66, 68.

The Roche Linear Array (LA) is a laboratory‐based assay that is not generally used in clinical practice but which enables the identification of 37 high‐risk HPV genotypes individually. PGMY09/11 primers are used to amplify the L1 region of the HPV genome using real‐time PCR. The resulting amplicons undergo reverse‐line blot hybridization to individually detect genotypes.^29^ Only the 14 high‐risk types that are identified by the Xpert HPV assay were included in this analysis.

### 2.4 Statistical analysis

Clinical and demographic information was summarized using descriptive statistics. Groups were compared using two‐sample *t*‐tests for continuous variables and chi‐squared or Fisher's exact tests for categorical variables. Overall and type‐specific HPV prevalence was calculated using only data from the general population. Frequency tables were used to determine HPV distributions by age group, HIV status, and cervical disease status. Diagnostic agreement between GX and LA was compared using percent overall agreement and Cohen's kappa statistic. We used multivariable logistic regression to determine which HPV genotypes were significantly associated with CIN2+ and CIN3+ (*P* < .05). By study design, women with CIN2+ and CIN3+ identified in both the general and the referral populations were included whereas women with <CIN2 or <CIN3 only from the general population were included in the analyses. This design was selected to obtain a sufficiently large number of women with cervical disease with the resources available.^30^ After selecting the significant genotypes from the multivariable logistic regression results, we calculated sensitivity and specificity comparing full vs restricted genotyping. Ninety‐five percent confidence intervals (CI) were calculated using the binomial estimates of the standard errors around these proportions. All statistical analyses were conducted using SAS 9.4. The data that support the findings of this study are available on request from the corresponding author. The data are not publicly available due to privacy or ethical restrictions.

## 3 Results

### 3.1 Demographic and clinical characteristics

Seven hundred and fifteen women were recruited at the community‐based clinic in Khayelitsha and 406 women from the referral colposcopy clinic. Of the 1121 women, 1118 had valid GX and LA results. Baseline demographic and clinical characteristics of the women recruited at the community‐based clinic are summarized by HIV status in Table 1. Our sample had similar proportions of women without HIV (382, 53.50%) and women living with HIV (332, 46.50%) by design. The prevalence of cervical intraepithelial neoplasia was higher for women living with HIV (17.02% CIN2+, 8.51% CIN3+) compared to women without HIV (5.29% CIN2+, 3.17% CIN3+). Women living with HIV were significantly younger (*P* < .001), had fewer children (*P* < .001), were less educated (*P* = .001), and had more prior cytology screenings (*P* = .006) than women without HIV. Of the women living with HIV, 265 (79.82%) reported currently taking antiretroviral medications. There were no differences observed for tobacco use (*P* = .954) and employment status (*P* = .740) by HIV status.

**Table 1:** Baseline demographic and clinical characteristics of 714 women recruited at a community clinic in Khayelitsha, South Africa (general population)

	Women without HIV (N = 382)		Women living with HIV (N = 332)		*P*‐value
	Mean or N	(SD) range or %	Mean or N	(SD) range or %	
Age	44.43	(9.31) 30‐65	40.73	(7.32) 30‐62	*P* < .0001
Parity	2.86	(1.55) 0‐12	2.25	(1.35) 0‐7	*P* < .0001
Age					
30‐39	130	34.03	171	51.51	*P* < .0001
40‐49	126	32.98	112	33.73	
50+	126	32.98	49	14.76	
Education					
None	6	1.57	2	0.60	*P* = .0014
Less than HS	61	15.97	34	10.24	
Some HS	74	19.37	57	17.17	
Finished 10	135	35.34	166	50.00	
HS graduate	106	27.75	73	21.99	
Tobacco					
Current	42	10.99	38	11.45	*P* = .9544
Former	20	5.24	16	4.82	
Never	320	83.77	278	83.73	
Employment					
Full‐time	110	28.80	87	26.20	*P* = .7404
Part‐time	35	9.16	32	9.64	
Unemployed	237	62.04	213	64.16	
Antiretrovirals					
Yes			265	79.82	—
No			67	20.1	
Cancer history					
Yes	1	0.26	0	0.00	*P* = .4183
No	380	99.48	332	100.00	
Do not know	1	0.26	0	0.00	
Prior cytology screening					
Yes	246	64.60	248	74.70	*P* = .0056
No	136	35.60	83	25.00	
Do not know	0	0.00	1	0.30	

### 3.2 HPV prevalence

#### 3.2.1 Overall

**Figure 1:**
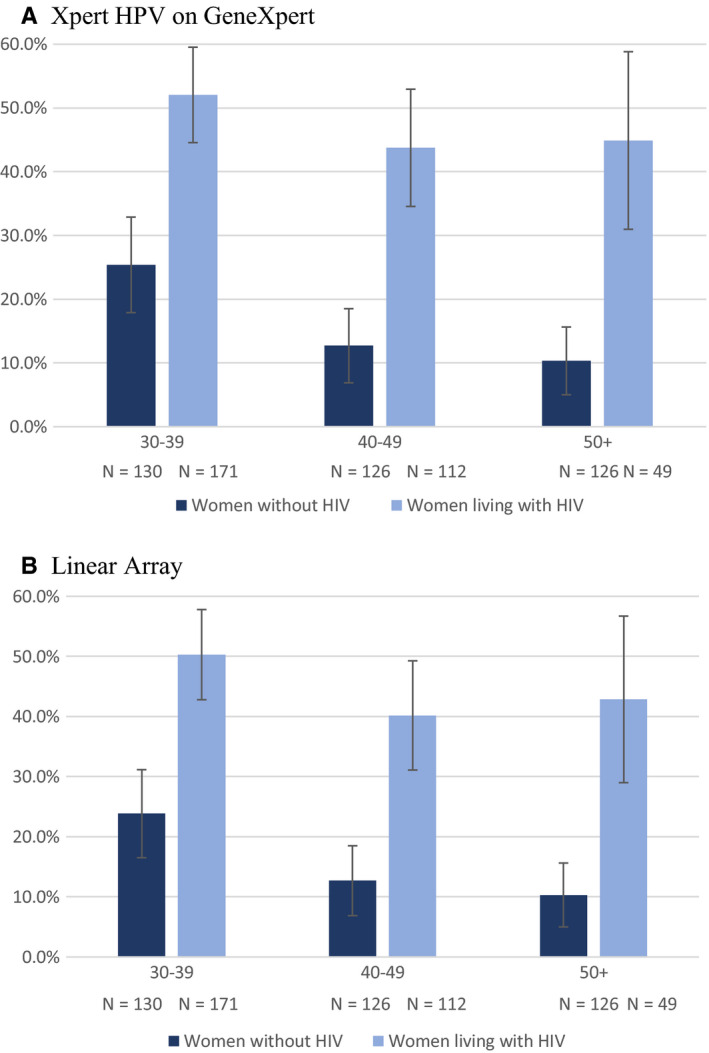
Prevalence of high risk HPV by HIV status and age group among 714 women in the general population recruited in Khayelitsha, South Africa. A, Xpert HPV on GeneXpert. B, Linear Array

For both assays, HPV prevalence for any type was higher regardless of age group for women living with HIV (48.19% [42.82%‐53.57%)] by GX, 45.78% [40.42%‐51.14%)] by LA) compared to women without HIV (16.23% [12.53%‐19.93%)] by GX, 15.71% [12.06%‐19.36%] by LA) (Figure 1).

#### 3.2.2 Type‐specific

Using GX in women living with HIV, the channel detecting HPV 31, 33, 35, 52, 58 (25.00%) was the most common followed by HPV 18, 45 (18.07%), HPV 39, 56, 66, 68 (10.84%), HPV 16 (10.54%), and HPV 51, 59 (6.63%). In women without HIV, the rankings were HPV 31, 33, 35, 52, 58 (8.12%) followed by HPV 39, 56, 66, 68 (3.66%); HPV 16 (3.40%); HPV 18, 45 (3.40%); and HPV 51, 59 (2.88%).

Using LA, the three most prevalent individual HPV types for women living with HIV were HPV 45 (10.24%), HPV 16 (9.94%), and HPV 18 (7.23%)/HPV 58 (7.23%). For women without HIV, HPV 16 (3.40%), HPV 51 (2.88%), and HPV 35 (2.09%)/HPV 52 (2.09%) were most prevalent.

#### 3.2.3 Multiple types

**Figure 2:**
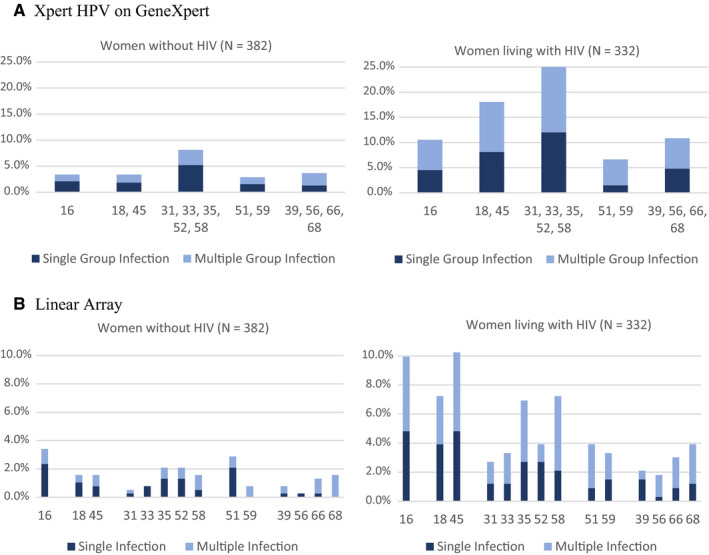
Prevalence of single and multiple high‐risk HPV infections by HIV status among 714 women in the general population recruited in Khayelitsha, South Africa infections. A, Xpert HPV on GeneXpert. B, Linear Array

Of the women identified as HPV‐positive by GX (N = 222) and LA (N = 212), multiple infections were more common among women living with HIV (35.63% by GX, 34.87% by LA) compared to women without HIV (25.81% by GX, 28.33% by LA) (Figure 2). Multiple infections were also more common among younger women: 30‐39 years (40.45%, 38.37%), 40‐49 years (32.65%, 33.33%), and 50+ years (22.73%, 23.81%) for GX and LA, respectively, in women living with HIV; and 30‐39 years (24.24%, 32.26%), 40‐49 years (37.50%, 31.25%), and 50+ years (15.38%, 15.38%) for GX and LA, respectively, in women without HIV.

### 3.3 Diagnostic concordance

The overall concordance between GX and LA for any of the 14 HPV genotypes was 93.56% (668 of 714 results), with a strong kappa value of 0.85 [0.81, 0.89]. Of the 222 women identified as HPV positive by GX, 194 (87.39%) were also deemed positive according to LA. Similarly, 194 (91.51%) of the 212 women identified as HPV positive by LA were also positive by GX. When the HPV genotypes were grouped following the five channels of GX, four of the five channel groups had strong kappa values: 0.86 [0.79, 0.94] for HPV16; 0.89 [0.83, 0.95] for HPV 18,45; 0.83 [0.77, 0.89] for HPV 31,33,35,52,58; 0.85 [0.76, 0.94] for HPV51,59. The channel detecting HPV 39, 56, 66, 68 had only a moderate kappa value of 0.72 [0.62, 0.83] compared to LA detection of these same genotypes.

**Figure 3:**
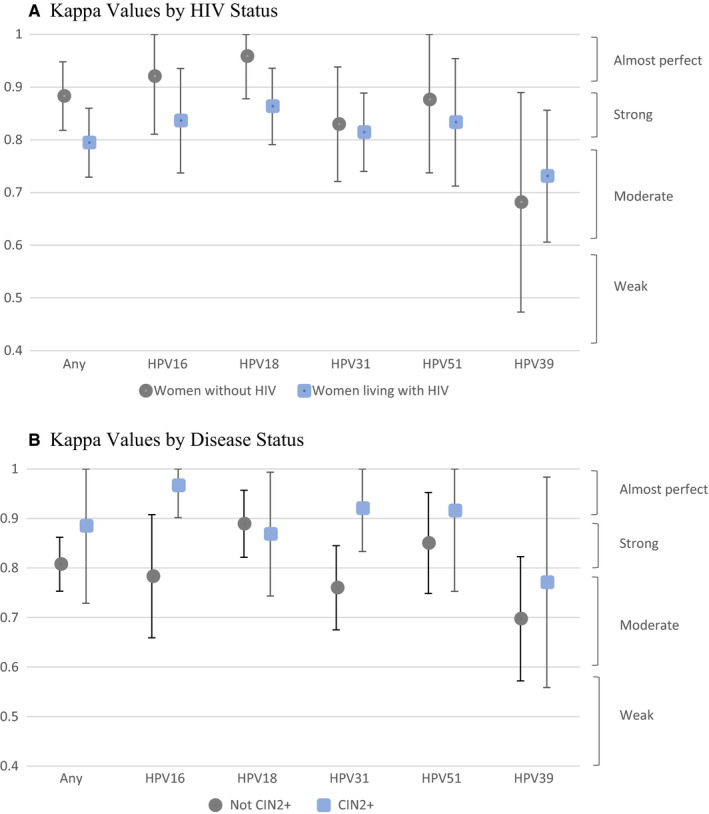
Diagnostic agreement between Linear Array and Xpert HPV on GeneXpert in detecting high‐risk HPV. A, Kappa Values by HIV Status. B, Kappa Values by Disease Status

Figure 3A presents kappa values by HIV status. Agreement for any HPV type was somewhat higher for women without HIV (96.86%, kappa 0.88 [0.82, 0.95]) than for women living with HIV (89.76%, kappa 0.79 [0.73, 0.86]). Kappas were higher for women without HIV than for women living with HIV across all HPV genotype groups except HPV 39, 56, 66, 68.

Figure 3B presents kappa values by cervical disease status. Agreement between the tests for any HPV genotype was higher among women with CIN2+ (97.37%, kappa 0.89 [0.73‐1.00]) than among women with <CIN2 (93.03%, kappa 0.81 [0.75‐0.86]). Agreement across tests among women with CIN2+ remained higher than that among women with <CIN2 for all HPV genotype groups except HPV 18, 45.

### 3.4 Selecting HPV genotypes to include

Table 2 shows results from univariable and multivariable logistic analyses associating individual HPV types (LA) or channels (GX) with CIN2+. Using GX, only the three channels detecting types 16, 18, 45, 31, 33, 35, 52, 58 were significantly (*P* < .05) associated with CIN2+ in the multivariable analysis regardless of HIV status. For LA, only HPV types 16, 18, 31, 33, 35, 52, and 58 were significantly associated with CIN2+ in multivariable analysis. HPV 59, which was significantly associated with CIN2+ in univariable analysis, was no longer associated with CIN2+ once the above‐mentioned HPV types were included in the model. In sum, both GX and LA assays identified the same seven HPV genotypes. The exception was HPV45 which was not significant as an individual type in the LA analysis but was included along with HPV 18 in one channel on GX. For the purpose of further analysis, we included HPV45 (due to its known links to adenocarcinoma) along with the seven consistently identified genotypes, in our calculations of selected typing. Full typing was defined as all five channels for GX and the 14 individual high‐risk HPV types for LA.

**Table 2:** Results of univariable and multivariable logistic regression analyses for individual high‐risk HPV genotypes/groups associated with cervical intraepithelial neoplasia grade 2 or greater (CIN2+)

	GeneXpert		Linear Array	
	Univariable odds ratio (95% CI)	Adjusted OR (95% CI)	Univariable odds ratio (95% CI)	Adjusted OR (95% CI)
Women without HIV				
ANY	51.991* (27.548‐98.121)		36.168* (20.422‐64.052)	
HPV16	27.993* (13.202‐59.356)	64.532* (27.905‐149.232)	24.491* (11.523‐52.052)	44.516* (19.801‐100.081)
HPV18+	4.896* (2.285‐10.492)	7.578* (2.789‐20.593)		
18			6.870* (2.338‐20.192)	13.125* (3.864‐44.586)
45			1.957 (0.543‐7.052)	2.668 (0.510‐13.945)
HPV31+	12.578* (7.200‐21.971)	25.745* (13.213‐50.166)		
31			9.044* (1.801‐45.402)	26.968* (5.073‐143.361)
33			7.475* (1.432‐39.030)	21.310* (3.770‐120.442)
35			16.273* (4.626‐57.239)	39.039* (10.453‐145.797)
52			6.284* (2.306‐17.130)	13.515* (4.432‐41.212)
58			5.525* (1.815‐16.819)	12.988* (3.801‐44.384)
HPV51+	2.402 (0.926‐6.229)			
51			1.973 (0.688‐5.660)	
59			7.475* (1.432‐39.030)	
HPV39+	2.176 (0.854‐5.540)			
39			0.962 (0.099‐9.336)	
56			2.898 (0.180‐46.683)	
66			0.720 (0.080‐6.500)	
68			1.751 (0.412‐7.437)	
Women living with HIV				
ANY	22.017* (11.413‐42.471)		23.430* (12.399‐44.273)	
HPV16	5.954* (3.375‐10.501)	10.156* (5.269‐19.575)	7.154* (3.927‐13.033)	12.039* (6.143‐23.594)
HPV18+	2.529* (1.579‐4.051)	2.709* (1.535‐4.781)		
18			2.409* (1.272‐4.564)	3.143* (1.453‐6.801)
45			2.041* (1.104‐3.774)	2.274* (1.070‐4.831)
HPV31+	7.913* (5.112‐12.249)	9.599* (5.885‐ 15.657)		
31			3.662* (1.250‐10.730)	5.352* (1.525‐18.784)
33			15.306* (4.545‐51.550)	25.799* (7.169‐92.838)
35			5.425* (2.845‐10.344)	7.325* (3.499‐15.338)
52			4.168* (1.691‐10.273)	9.733* (3.617‐26.192)
58			4.083* (2.144‐7.776)	5.108* (2.393‐10.905)
HPV51+	1.981* (1.007‐3.899)			
51			2.150 (0.921‐5.019)	
59			1.616 (0.595‐4.389)	
HPV39+	3.069* 1.801‐5.230			
39			1.605 (0.458‐5.627)	
56			2.013 (0.533‐7.604)	
66			2.045 (0.791‐5.287)	
68			1.431 (0.541‐ 3.783)	

* *P* < .05.

Results from the univariable and multivariable logistic regression analyses for CIN3+ were similar and are included as Table S1.

### 3.5 Comparison of full vs restricted genotyping

After selecting the genotypes that were significantly associated with CIN2+ from the multivariable logistic regression results, we calculated sensitivity and specificity comparing full vs restricted genotyping. For both assays, there was only a slight decrease in sensitivity for restricted vs full genotyping. For women living with HIV, the sensitivity to detect CIN2+ with full typing was 93.60% [88.85%‐96.76%] by GX and 93.02% [88.13%‐96.34%] by LA, which decreased to 90.70% [85.33%‐94.59%] by GX and 88.95% [83.29%‐93.22%] by LA for restricted genotyping. For women without HIV, the sensitivity to detect CIN2+ with full typing was 88.71% [81.78%‐93.69%] by GX and 83.87% [76.19%‐89.86%] by LA, which decreased to 87.10% [79.89%‐92.44%] by GX and 79.03% [70.81%‐85.85%] by LA for restricted genotyping (Table 3).

**Table 3:** Sensitivity to detect cervical intraepithelial neoplasia grade 2 or worse (CIN2+) with selected typing vs full typing using Xpert HPV on GeneXpert or Linear Array

	Sensitivity	Lower 95%	Upper 95%	Specificity	Lower 95%	Upper 95%
Xpert HPV on GeneXpert						
Women without HIV						
Any (all five channels)	88.71	81.78	93.69	86.87	82.93	90.19
16 (1 channel)	41.94	33.14	51.13	97.49	95.28	98.84
16, 18, 45 (2 channels)	54.03	44.85	63.01	94.41	91.50	96.55
16, 18, 45, 31, 33, 35, 52, 58 (3 channels)	87.10	79.89	92.44	89.66	86.04	92.62
Women living with HIV						
Any (all five channels)	93.60	88.85	96.76	60.07	54.00	65.93
16 (1 channel)	30.81	24.01	38.29	93.04	89.34	95.76
16, 18, 45 (2 channels)	55.81	48.06	63.37	79.49	74.20	84.12
16, 18, 45, 31, 33, 35, 52, 58 (3 channels)	90.70	85.33	94.59	67.77	61.87	73.27
Linear array						
Women without HIV						
Any	83.87	76.19	89.86	87.43	83.54	90.68
16	38.71	30.10	47.87	97.49	95.28	98.84
16, 18	45.16	36.21	54.35	96.09	93.53	97.85
16, 18, 45	48.39	39.32	57.53	94.41	91.50	96.55
16, 18, 45, 31	53.23	44.06	62.24	93.85	90.84	96.11
16, 18, 45, 31, 33	56.45	47.26	65.33	93.30	90.10	95.66
16, 18, 45, 31, 33, 35	66.13	57.09	74.38	93.02	89.86	95.43
16, 18, 45, 31, 33, 35, 52	73.39	64.70	80.92	91.90	88.57	94.51
16, 18, 45, 31, 33, 35, 52, 58	79.03	70.81	85.82	90.78	87.30	93.57
Women living with HIV						
Any	93.02	88.13	96.34	63.74	57.73	69.45
16	30.81	23.91	37.71	94.14	90.66	96.61
16, 18	43.02	35.51	50.78	87.91	83.44	91.53
16, 18, 45	53.49	45.74	61.12	82.05	76.97	86.42
16, 18, 45, 31	56.98	49.22	64.49	80.22	74.99	84.78
16, 18, 45, 31, 33	66.28	58.69	73.30	79.49	74.20	84.12
16, 18, 45, 31, 33, 35	76.74	69.71	82.84	76.56	71.07	81.45
16, 18, 45, 31, 33, 35, 52	82.56	76.05	87.91	74.73	69.13	79.77
16, 18, 45, 31, 33, 35, 52, 58	88.95	83.29	93.22	71.43	65.67	76.71

Sensitivity was slightly higher using GX than LA. In 11 women with CIN2+ who did not have one of the 14 genotypes detected by LA, but who did have them detected by GX, two were positive on the HPV 16 channel, two on the HPV18,45 channel, and nine positives on the HPV 31, 33, 35, 52, 58 channel (2 cases overlapped with the HPV 18, 45 channel). Nine of the 11 were reported as “no isolates” on LA, one was HPV 53 and one was IS39. In four women with CIN2+ who did not have one of the 14 genotypes detected by GX, but who did have them detected by LA, the HPV genotypes detected in LA were: 45; 16,83; 68,70; and 51, 53, 62, 71, 82, 83.

Specificity estimates improved with restricted compared to full genotyping. Specificity for full genotyping among women living with HIV was 60.07% [54.00%‐65.93%] for GX and 63.74% [57.73%‐69.45%] for LA. Specificity increased to 67.77% [61.87%‐73.27%] by GX and 71.43% [65.67%‐76.71%] by LA for restricted genotyping. For women without HIV, specificity was 86.87% [82.93%‐90.19%] for GX and 87.43% [83.54%‐90.68%] for LA. Specificity increased to 89.66% [86.04%‐92.62%] by GX and 90.78% [87.30%‐93.56%] by LA for restricted genotyping (Table 3).

In contrast to the marginal reduction in sensitivity moving from including all 14 high‐risk types to the eight selected types, selecting only HPV types 16 and 18 led to an appreciable drop in sensitivity. In women without HIV, sensitivity for full vs restricted vs 16,18 genotyping was 83.87%, 79.03%, and 45.16%, respectively. In women living with HIV, sensitivity for full vs restricted vs 16, 18 genotyping was 93.02%, 88.95%, and 43.02%, respectively. Utilizing the data from women enrolled in the screening population, the positive predictive values (PPV) for full vs restricted vs 16, 18 genotyping were 23.73%, 29.79%, and 26.32% for women without HIV and 34.87%, 38.58% and 40.00% for women living with HIV.

Sensitivity estimates were slightly higher and specificity estimates lower for detection of CIN3+ compared to CIN2+ but the benefits of restricted compared to full genotyping were similar (Table S2).

## 4 Discussion

To optimize the clinical performance of HPV testing in screening programs, our results indicate that only HPV 16, 18, 45, 31, 33, 35, 52, and 58 should be tested for. Although HPV 45 was not significantly associated with our disease endpoint, CIN2+, we include this genotype since it is the third most commonly identified genotype in invasive cervical carcinoma.^25^ Lack of significance in our analysis may be attributed to the small number of cases of invasive carcinoma in our sample. In this analysis, we observed that restricted genotyping, as compared to full genotyping, resulted in only marginal reductions in sensitivity but sizeable improvements in specificity. Since the inclusion of HPV 51, 59, 39, 56, 66, 68 when testing did not appreciably improve clinical performance, these genotypes could be excluded when HPV testing in high‐burden low‐resource settings where the capacity to treat or follow‐up HPV‐positive women is limited. Genotype‐restriction was even more effective for women living with HIV compared to women without HIV, with a greater increase in specificity observed moving from full to restricted genotyping. However, the highest specificity achieved with restricted genotyping for women living with HIV (67.77% GX, 71.43% LA) was still considerably lower than that of HIV negative women (89.66% GX, 90.78% LA). While improvements have been made, there is still a need to further explore mechanisms to improve specificity for women living with HIV, especially in settings such as South Africa which had an estimated HIV prevalence of 18.8% in 2017 (4th highest in the world).^2^

We examined whether the inclusion of only types 16 and 18 might be a simpler approach to HPV type selection. We found that restricting only to these types led to an unacceptable decline in sensitivity. Positive predictive value in the group with types 16 and 18 was not appreciably better than the PPV in the group of eight types that we selected.

In our analysis, we have additionally shown that point‐of‐care HPV DNA testing is a robust option for primary cervical cancer screening in limited‐resource settings like South Africa. The point‐of‐care GX used in South Africa has the benefit of rapid same‐day results and minimal lab infrastructure/personnel. In this analysis we found it to have equivalent clinical performance characteristics as laboratory‐based LA.^31^ Concordance between the two assays was excellent (94%), with a kappa of 0.88 in women without HIV and 0.79 in women living with HIV. Furthermore, this analysis confirms the validity of grouping HPV genotypes into the five separate channels offered by GX. The seven individual high‐risk HPV genotypes identified as significantly associated with CIN2+ by LA aligned with the same genotypes included in the first three channels of GX. Since the carcinogenic behavior of each HPV type is closely related to its phylogenic category, it is not surprising that these seven HPV types belong to the higher risk species related to cervical cancer: alpha‐9 (HPV 16, 31, 33, 35, 52, 58) and alpha‐7 (HPV 18).^32^ In particular, HPV35 was highly correlated to CIN2+/CIN3+ within our sample, which is consistent with recent studies that have found a strong link to cervical carcinogenesis for women of African ancestry with HPV35.^33^

The GX platform is a pragmatic choice for South Africa given its current widespread usage across the country for tuberculosis testing^34^ which offers potential leverage to utilize the same instruments for HPV screening. The global placements of the GX system now exceed 23 000 instruments, a large portion of which are in low resource settings (where TB is common), including Africa, Asia, and Latin America.^35^ In addition, GX HPV testing has met international performance standards for use in primary cervical cancer screening.^30^ Several countries have piloted the GX HPV test including South Africa,^36^ Cameroon,^37^ UK,^30^ Zambia,^38^ Saudia Arabia, and the USA.^39^, ^40^ Sensitivity rates in detecting CIN2+ ranged from 63.2% to 98.7% and specificity ranged from 42.6% to 90.3%. In our study, restricting HPV genotypes allowed us to achieve specificity rates that exceed estimates reported in most studies and should be considered for improving test performance. We observed only marginally lower specificity with GX compared to LA consistent with slightly higher sensitivity in GX compared to LA. It is possible that cross‐reactivity with low‐risk types or amplification of low signal when HPV is detected in pooled channels may contribute to these results.^41^

There are a few limitations to our study. First, we used disease endpoints (CIN2+/CIN3+) in our analysis but realize that not all of these cervical lesions will progress to cancer. Some researchers criticize the use of CIN2+ due to lack of reproducibility and the higher likelihood of these lesions regressing compared to CIN3+.^7^, ^36^ However, most published studies have used CIN2+ as an endpoint to have the international performance standards, which allows us to make meaningful comparisons.^42^ Nonetheless, our comparison of CIN2+ and CIN3+ showed similar results and the same conclusions about the clinical performance of GX and LA were drawn from either endpoint. Another limitation was that our sample size was small. Due to limited resources, we were unable to obtain sufficient cases of CIN2+ from the general screening population. Therefore, we needed to sample patients from a colposcopy referral clinic to obtain sufficient numbers of women with cervical disease for our analysis. Therefore, our sensitivity calculations included patients from the screening and referral populations, while specificity was calculated using only the screening population. This enrichment approach is supported in the recommendations for HPV test validation.^30^

The landscape of cervical cancer prevention is rapidly changing and on the horizon is primary screening with HPV DNA testing in resource‐constrained settings.^2^ Restricting the definition of screen‐positive to women with HPV genotypes 16, 18, 45, 31, 33, 35, 52, or 58 substantially improves the specificity of screening, while producing only minor reductions in sensitivity compared to inclusion of all 14 HPV genotypes conventionally included as high risk in HPV assays. Using the GX assay, genotype restriction can be easily accommodated given the existing channels. The lack of population benefit of testing for some of the HPV genotypes typically classified as “high‐risk” has been previously noted.^43^ Comparison of the distribution of individual high‐risk genotypes identified using LA did detect other HPV groupings that appreciably improved specificity on the groupings of the GX assay. Furthermore, comparable performance between LA and GX shows that HPV screening can be done at point‐of‐care with considerably less demand for resources. Point‐of‐care testing with GX is feasible and restricted genotyping can maintain sensitivity while improving specificity. As such, the next step is to integrate HPV testing into cervical cancer screening programs to improve the health of women.

## Conflict of Interest

LJ, RS, ZM, AW, RB, AT, JM, WYT, LD, and LK have no conflicts to declare. DP and SC declare that they receive salary, benefits, and equity from Danaher Corp. Cepheid is a wholly owned entity of Danaher Corporation. TW declares that he serves as a consultant in clinical trial design and as an expert pathologist for HPV vaccine and/or diagnostic trials for Becton, Dickinson and Company, BD Life Sciences – Diagnostic Systems, Roche, and Inovio Pharmaceuticals and as a speaker for Roche and Becton, Dickinson and Company, BD Life Sciences – Diagnostic Systems.

## Author Contributions

LK, TW, and LD designed the study. RS, RB, and LD recruited the participants and did the clinical follow‐up. DP and SC provided technical assistance on human papillomavirus testing with Xpert. ZM and AW conducted human papillomavirus testing with Linear Array. LJ, LK, and WYT undertook the statistical analyses. LJ and LK wrote the first draft. JM, DP, and TW provided crucial input in data interpretation. All authors reviewed the content of the final version of the manuscript.

## Supporting information

Table S1‐S2

## Data Availability

The data that support the findings of this study are available on request from the corresponding author. The data are not publicly available due to privacy or ethical restrictions.

## References

[1] Arbyn M , Snijders PJ , Meijer CJ , et al. Which high‐risk HPV assays fulfil criteria for use in primary cervical cancer screening? Clin Microbiol Infect. 2015;21(9):817–826. 25936581 10.1016/j.cmi.2015.04.015

[2] Kuhn L , Denny L . The time is now to implement HPV testing for primary screening in low resource settings. Prevent Med. 2017;98:42–44. 10.1016/j.ypmed.2016.12.030 PMC5578476 28279263

[3] Domgue JF , Valea FA . Is it relevant to keep advocating visual inspection of the cervix with acetic acid for primary cervical cancer screening in limited‐resource settings? J Glob Oncol. 2018;4:1–5. 10.1200/JGO.17.00048 PMC6180765 30241142

[4] Sahasrabuddhe VV , Parham GP , Mwanahamuntu MH , Vermund SH . Cervical cancer prevention in low‐ and middle‐income countries: feasible, affordable, essential. Cancer Prev Res. 2012;5(1):11–17. 10.1158/1940-6207.CAPR-11-0540 PMC3586242 22158053

[5] Kelly H , Mayaud P , Segondy M , Pant Pai N , Peeling RW . A systematic review and meta‐analysis of studies evaluating the performance of point‐of‐care tests for human papillomavirus screening. Sex Transm Infect. 2017;93(S4):S36–S45. 29223961 10.1136/sextrans-2016-053070

[6] Tota JE , Bentley J , Blake J , et al. Introduction of molecular HPV testing as the primary technology in cervical cancer screening: acting on evidence to change the current paradigm. Prevent Med. 2017;98:5–14. 10.1016/j.ypmed.2016.11.029 28279264

[7] Schiffman M , Wentzensen N , Wacholder S , Kinney W , Gage JC , Castle PE . Human papillomavirus testing in the prevention of cervical cancer. J Natl Cancer Inst. 2011;103(5):368–383. 21282563 10.1093/jnci/djq562 PMC3046952

[8] Sankaranarayanan R , Nessa A , Esmy PO , Dangou JM . Visual inspection methods for cervical cancer prevention. Best Pract Res Clin Obstet Gynaecol. 2012;26(2):221–32. 22075441 10.1016/j.bpobgyn.2011.08.003

[9] Arbyn M , Sasieni P , Meijer CJ , Clavel C , Koliopoulos G , Dillner J . Chapter 9: clinical applications of HPV testing: a summary of meta‐analyses. Vaccine. 2006;24(Suppl 3):S78–S89. 10.1016/j.vaccine.2006.05.117 16950021

[10] Arbyn M , Ronco G , Anttila A , et al. Evidence regarding human papillomavirus testing in secondary prevention of cervical cancer. Vaccine. 2012;30(Suppl 5):F88–F99. 23199969 10.1016/j.vaccine.2012.06.095

[11] Cuzick J , Arbyn M , Sankaranarayanan R , et al. Overview of human papillomavirus‐based and other novel options for cervical cancer screening in developed and developing countries. Vaccine. 2008;26(Suppl 10):K29–K41. 18847555 10.1016/j.vaccine.2008.06.019

[12] Mapanga W , Girdler‐Brown B , Feresu SA , Chipato T , Singh E . Prevention of cervical cancer in HIV‐seropositive women from developing countries through cervical cancer screening: a systematic review. Syst Rev. 2018;7(1):198. 30447695 10.1186/s13643-018-0874-7 PMC6240280

[13] WHO Comprehensive Cervical Cancer Control: A guide to essential practice. 2nd ed. Geneva, Switzerland: World Health Organization ; 2014. 25642554

[14] Pileggi C , Flotta D , Bianco A , Nobile CG , Pavia M . Is HPV DNA testing specificity comparable to that of cytological testing in primary cervical cancer screening? Results of a meta‐analysis of randomized controlled trials. Int J Cancer. 2014;135(1):166–177. 24302411 10.1002/ijc.28640

[15] Seay JS , Kobetz E . Optimizing cervical cancer screening and triage in low‐resource settings. J Glob Oncol. 2018;4:1–2. 10.1200/JGO.18.00192 PMC6223538 30252572

[16] Wentzensen N , Schiffman M , Palmer T , Arbyn M . Triage of HPV positive women in cervical cancer screening. J Clin Virol. 2016;76(Suppl 1):S49–S55. 26643050 10.1016/j.jcv.2015.11.015 PMC4789103

[17] Catarino R , Petignat P , Dongui G , Vassilakos P . Cervical cancer screening in developing countries at a crossroad: emerging technologies and policy choices. World J Clin Oncol. 2015;6(6):281–290. 26677441 10.5306/wjco.v6.i6.281 PMC4675913

[18] Poli UR , Gowrishankar S , Swain M , Jeronimo J . Triage of women testing positive with the careHPV test on self‐collected vaginal samples for cervical cancer screening in a low‐resource setting. J Glob Oncol. 2018;4:1–7. 10.1200/JGO.2016.008078 PMC6180797 30241206

[19] Wang M , Hu S , Zhao S , et al. Accuracy of triage strategies for human papillomavirus DNA‐positive women in low‐resource settings: a cross‐sectional study in China. Chin J Cancer Res. 2017;29(6):496–509. 29353972 10.21147/j.issn.1000-9604.2017.06.04 PMC5775023

[20] Tota JE , Bentley J , Blake J , et al. Approaches for triaging women who test positive for human papillomavirus in cervical cancer screening. Prevent Med. 2017;98:15–20. 10.1016/j.ypmed.2016.11.030 28279257

[21] Jeronimo J , Castle PE , Temin S , et al. Secondary prevention of cervical cancer: ASCO resource‐stratified clinical practice guideline. J Glob Oncol. 2017;3(5):635–657. 29094101 10.1200/JGO.2016.006577 PMC5646891

[22] Gravitt PE , Coutlee F , Iftner T , Sellors JW , Quint WG , Wheeler CM . New technologies in cervical cancer screening. Vaccine. 2008;26(Suppl 10):K42–K52. 18847556 10.1016/j.vaccine.2008.05.002

[23] International Agency for Research on Cancer (IARC) . IARC Monographs on the Evaluation of Carcinogenic Risks to Humans. Volume 90. Human Papillomaviruses. Lyon, France: IARC; 2007.

[24] Smith JS , Lindsay L , Hoots B , et al. Human papillomavirus type distribution in invasive cervical cancer and high‐grade cervical lesions: a meta‐analysis update. Int J Cancer. 2007;121(3):621–632. 17405118 10.1002/ijc.22527

[25] de Sanjose S , Quint WG , Alemany L , et al. Human papillomavirus genotype attribution in invasive cervical cancer: a retrospective cross‐sectional worldwide study. Lancet Oncol. 2010;11(11):1048–1056. 20952254 10.1016/S1470-2045(10)70230-8

[26] Li N , Franceschi S , Howell‐Jones R , Snijders PJ , Clifford GM . Human papillomavirus type distribution in 30,848 invasive cervical cancers worldwide: variation by geographical region, histological type and year of publication. Int J Cancer. 2011;128(4):927–935. 20473886 10.1002/ijc.25396

[27] Serrano B , de Sanjose S , Tous S , et al. Human papillomavirus genotype attribution for HPVs 6, 11, 16, 18, 31, 33, 45, 52 and 58 in female anogenital lesions. Eur J Cancer. 2015;51(13):1732–1741. 26121913 10.1016/j.ejca.2015.06.001

[28] Sellors JW , Sankaranarayanan R . Colposcopy and treatment of cervical intraepithelial neoplasia: a beginners’ manual. France: International Agency for Research on Cancer; 2003. 33689255

[29] Coutlee F , Rouleau D , Petignat P , et al. Enhanced detection and typing of human papillomavirus (HPV) DNA in anogenital samples with PGMY primers and the Linear array HPV genotyping test. J Clin Microbiol. 2006;44(6):1998–2006. 16757590 10.1128/JCM.00104-06 PMC1489445

[30] Arbyn M , Depuydt C , Benoy I , et al. VALGENT: a protocol for clinical validation of human papillomavirus assays. J Clin Virol. 2016;76(Suppl 1):S14–S21. 26522865 10.1016/j.jcv.2015.09.014

[31] Xu L , Ostrbenk A , Poljak M , Arbyn M . Assessment of the Roche Linear Array HPV genotyping test within the VALGENT framework. J Clin Virol. 2018;98:37–42. 29241150 10.1016/j.jcv.2017.12.001

[32] Schiffman M , Clifford G , Buonaguro FM . Classification of weakly carcinogenic human papillomavirus types: addressing the limits of epidemiology at the borderline. Infect Agent Cancer. 2009;4:8. 19486508 10.1186/1750-9378-4-8 PMC2694995

[33] Pinheiro M , Gage JC , Clifford GM , et al. Association of HPV35 with cervical carcinogenesis among women of African ancestry: evidence of viral‐host interaction with implications for disease intervention. Int J Cancer. 2020 10.1002/ijc.33033 PMC11090644 32363580

[34] Meyer‐Rath G , Schnippel K , Long L , et al. The impact and cost of scaling up GeneXpert MTB/RIF in South Africa. PLoS One. 2012;7(5):e36966. 22693561 10.1371/journal.pone.0036966 PMC3365041

[35] Cepheid Announces FleXible Cartridge Program. https://www.prnewswire.com/news‐releases/cepheid‐announces‐flexible‐cartridge‐program‐300831763.html. Accessed February 19, 2020.

[36] Mittal S , Ghosh I , Banerjee D , et al. Reproducibility of cervical intraepithelial neoplasia diagnosis on histological review of cervical punch biopsies from a visual inspection with acetic acid and HPV detection‐based screening program. Int J Gynaecol Obstet. 2014;126(3):227–231. 24947603 10.1016/j.ijgo.2014.03.037

[37] Kunckler M , Schumacher F , Kenfack B , et al. Cervical cancer screening in a low‐resource setting: a pilot study on an HPV‐based screen‐and‐treat approach. Cancer Med. 2017;6(7):1752–1761. 28580596 10.1002/cam4.1089 PMC5504339

[38] Chibwesha CJ , Frett B , Katundu K , et al. Clinical performance validation of 4 point‐of‐care cervical cancer screening tests in HIV‐infected women in Zambia. J Low Genit Tract Dis. 2016;20(3):218–223. 27030883 10.1097/LGT.0000000000000206 PMC4920696

[39] Castle PE , Smith KM , Davis TE , et al. Reliability of the Xpert HPV assay to detect high‐risk human papillomavirus DNA in a colposcopy referral population. Am J Clin Pathol. 2015;143(1):126–133. 25511151 10.1309/AJCP4Q0NSDHWIZGU

[40] Einstein MH , Smith KM , Davis TE , et al. Clinical evaluation of the cartridge‐based GeneXpert human papillomavirus assay in women referred for colposcopy. J Clin Microbiol. 2014;52(6):2089–2095. 24719440 10.1128/JCM.00176-14 PMC4042758

[41] Preisler S , Rebolj M , Ejegod DM , Lynge E , Rygaard C , Bonde J . Cross‐reactivity profiles of hybrid capture II, cobas, and APTIMA human papillomavirus assays: split‐sample study. BMC Cancer. 2016;16:510. 27439470 10.1186/s12885-016-2518-4 PMC4955240

[42] Meijer CJ , Berkhof J , Castle PE , et al. Guidelines for human papillomavirus DNA test requirements for primary cervical cancer screening in women 30 years and older. Int J Cancer. 2009;124(3):516–520. 18973271 10.1002/ijc.24010 PMC2789446

[43] Schiffman M , Khan MJ , Solomon D , et al. A study of the impact of adding HPV types to cervical cancer screening and triage tests. J Natl Cancer Inst. 2005;97(2):147–150. 15657345 10.1093/jnci/dji014

