# Supplementary material for: Selecting human papillomavirus genotypes to optimize the performance of screening tests among South African women
Source: Cancer Med. 2020 Jul 24;9(18):6813–24. doi: 10.1002/cam4.3329 (PMC7520316; doi:10.1002/cam4.3329)
Supplement: Supplementary file 1 — Table S1‐S2 [file CAM4-9-6813-s001.docx]

**Supplementary Material**

Table S1. Results of univariable and multivariable logistic regression analyses for individual high-risk HPV types/groups associated with cervical intraepithelial neoplasia grade 3 or greater (CIN3+)

|  | **Women without HIV** | | | |
| --- | --- | --- | --- | --- |
|  | **GeneXpert** | | **Linear Array** | |
|  | **Univariable**  **Odds Ratio**  **(95% CI)** | **Adjusted OR**  **(95% CI)** | **Univariable**  **Odds Ratio**  **(95% CI)** | **Adjusted OR**  **(95% CI)** |
| ANY | 91.634*  (38.031 - 220.788) |  | 44.719*  (22.701 - 88.091) |  |
| HPV16 | 31.967*  (15.119 - 67.591) | 76.495*  (31.711 - 184.527) | 28.069*  (13.259 - 59.423) | 51.508*  (22.489 - 117.971) |
| HPV18+ | 3.957*  (1.687 - 9.283) | 7.365*  (2.235 - 24.276) |  |  |
| 18 |  |  | 5.874*  (1.820 - 18.953) | 13.463*  (3.477 - 52.123) |
| 45 |  |  | 2.000  (0.491 - 8.151) | 4.163  (0.789 - 21.958) |
| HPV31+ | 11.735*  (6.561 - 20.990) | 29.063*  (13.753 - 61.417) |  |  |
| 31 |  |  | 12.550*  (2.490 - 63.241) | 41.053*  (7.589 - 222.080) |
| 33 |  |  | 10.340*  (1.974 - 54.180) | 32.421*  (5.636 - 186.503) |
| 35 |  |  | 7.736*  (2.527 - 23.678) | 20.976*  (6.150 - 71.540) |
| 52 |  |  | 5.645*  (1.908 - 16.700) | 9.802*  (2.613 - 36.772) |
| 58 |  |  | 5.874*  (1.820 - 18.953) | 13.314*  (3.412 - 51.945) |
| HPV51+ | 2.455  (0.869 - 6.939) |  |  |  |
| 51 |  |  | 2.254  (0.737 - 6.893) |  |
| 59 |  |  | 8.180*  (1.475 - 45.367) |  |
| HPV39+ | 2.226  (0.801 - 6.185) |  |  |  |
| 39 |  |  | <0.001  (<0.001 - >999.999) |  |
| 56 |  |  | 3.967  (0.246 - 64.029) |  |
| 66 |  |  | <0.001  (<0.001 - >999.999) |  |
| 68 |  |  | 1.588  (0.303 - 8.315) |  |
|  | **Women living with HIV** | | | |
|  | **GeneXpert** | | **Linear Array** | |
|  | **Univariable**  **Odds Ratio**  **(95% CI)** | **Adjusted OR**  **(95% CI)** | **Univariable**  **Odds Ratio**  **(95% CI)** | **Adjusted OR**  **(95% CI)** |
| ANY | 25.516*  (10.104 - 64.432) |  | 17.240*  (8.094 - 36.721) |  |
| HPV16 | 5.907*  (3.379 - 10.328) | 8.006*  (4.145 - 15.466) | 6.426*  **(**3.639 - 11.348) | 8.813*  (4.585 - 16.938) |
| HPV18+ | 1.402  **(**0.813 - 2.415) | 1.266  (0.664 - 2.414) |  |  |
| 18 |  |  | 2.316*  (1.139 - 4.710) | 3.210*  (1.339 - 7.693) |
| 45 |  |  | 0.808  (0.371 - 1.759) | 0.649  (0.255 - 1.655) |
| HPV31+ | 7.650*  (4.684 - 12.493) | 8.929*  **(**5.153 - 15.471) |  |  |
| 31 |  |  | 4.014*  (1.359 - 11.853) | 4.986*  (1.336 - 18.609) |
| 33 |  |  | 6.390*  (2.504 - 16.310) | 11.166*  (3.768 - 33.091) |
| 35 |  |  | 5.213*  **(**2.645 - 10.276) | 6.296*  (2.856 - 13.880) |
| 52 |  |  | 3.053*  (1.282 - 7.266) | 5.886*  (5.886 - 15.736) |
| 58 |  |  | 4.711*  (2.326 - 9.538) | 5.251*  (2.274 - 12.124) |
| HPV51+ | 1.627  (0.752 - 3.520) |  |  |  |
| 51 |  |  | 1.445  (0.528 - 3.952) |  |
| 59 |  |  | 1.272  (0.384 - 4.221) |  |
| HPV39+ | 1.774  (0.960 - 3.279) |  |  |  |
| 39 |  |  | 0.946  (0.188 - 4.760) |  |
| 56 |  |  | 0.468  (0.056 - 3.935) |  |
| 66 |  |  | 2.590  (0.916 - 7.325) |  |
| 68 |  |  | 0.646  (0.180 - 2.311) |  |

*p<.05

Table S2: Sensitivity to detect cervical intraepithelial neoplasia grade 3 or worse (CIN3+) with selected typing versus full typing using Xpert HPV on GeneXpert or Linear Array

|  | **Sensitivity** | **Lower 95%** | **Upper 95%** | **Specificity** | **Lower 95%** | **Upper 95%** |
| --- | --- | --- | --- | --- | --- | --- |
| **Xpert HPV on GeneXpert** |  |  |  |  |  |  |
| **Women without HIV** |  |  |  |  |  |  |
| Any (all 5 channels) | 93.55 | 86.48 | 97.60 | 86.34 | 82.39 | 89.69 |
| 16 (1 channel) | 47.31 | 36.86 | 57.94 | 97.27 | 95.03 | 98.68 |
| 16, 18, 45 (2 channels) | 55.91 | 45.24 | 66.20 | 94.26 | 91.36 | 96.41 |
| 16, 18, 45, 31, 33, 35, 52, 58 (3 channels) | 91.40 | 83.75 | 96.21 | 89.07 | 85.42 | 92.08 |
| **Women living with HIV** |  |  |  |  |  |  |
| Any (all 5 channels) | 95.28 | 89.33 | 98..45 | 55.81 | 50.00 | 61.51 |
| 16 (1 channel) | 36.79 | 27.63 | 46.71 | 91.03 | 87.22 | 94.01 |
| 16, 18, 45 (2 channels) | 57.55 | 47.57 | 67.09 | 75.42 | 70.15 | 80.17 |
| 16, 18, 45, 31, 33, 35, 52, 58 (3 channels) | 93.40 | 86.87 | 97.30 | 63.46 | 57.74 | 68.91 |
| **Linear Array** |  |  |  |  |  |  |
| **Women without HIV** |  |  |  |  |  |  |
| Any | 87.10 | 78.55 | 93.15 | 86.89 | 82.99 | 90.17 |
| 16 | 44.09 | 33.80 | 54.76 | 97.27 | 95.03 | 98.68 |
| 16, 18 | 49.46 | 38.93 | 60.03 | 95.90 | 93.33 | 97.69 |
| 16, 18, 45 | 52.69 | 42.06 | 63.14 | 94.26 | 91.36 | 96.41 |
| 16, 18, 45, 31 | 59.14 | 48.46 | 69.23 | 93.72 | 90.72 | 95.98 |
| 16, 18, 45, 31, 33 | 63.44 | 52.81 | 73.19 | 93.17 | 90.08 | 95.53 |
| 16, 18, 45, 31, 33, 35 | 72.04 | 61.78 | 80.86 | 92.35 | 89.13 | 94.86 |
| 16, 18, 45, 31, 33, 35, 52 | 77.42 | 67.58 | 85.45 | 91.26 | 87.88 | 93.94 |
| 16, 18, 45, 31, 33, 35, 52, 58 | 82.80 | 73.57 | 89.83 | 90.16 | 86.64 | 93.02 |
| **Women living with HIV** |  |  |  |  |  |  |
| Any | 92.45 | 85.67 | 96.69 | 58.47 | 52.68 | 64.10 |
| 16 | 36.79 | 27.63 | 46.71 | 91.69 | 87.98 | 94.55 |
| 16, 18 | 48.11 | 38.30 | 58.03 | 85.38 | 80.88 | 89.17 |
| 16, 18, 45 | 53.77 | 43.82 | 63.51 | 77.08 | 71.91 | 81.70 |
| 16, 18, 45, 31 | 57.55 | 47.57 | 67.09 | 75.42 | 70.15 | 80.17 |
| 16, 18, 45, 31, 33 | 66.98 | 57.18 | 75.81 | 74.42 | 69.10 | 79.25 |
| 16, 18, 45, 31, 33, 35 | 77.36 | 68.21 | 84.92 | 71.76 | 66.31 | 76.78 |
| 16, 18, 45, 31, 33, 35, 52 | 83.96 | 75.57 | 90.37 | 69.10 | 63.55 | 74.28 |
| 16, 18, 45, 31, 33, 35, 52, 58 | 89.62 | 82.19 | 94.70 | 66.11 | 60.46 | 71.44 |
